# Computed tomography of the spleen: how to interpret the hypodense lesion

**DOI:** 10.1007/s13244-012-0202-z

**Published:** 2012-12-04

**Authors:** Christoph A. Karlo, Paul Stolzmann, Richard K. Do, Hatem Alkadhi

**Affiliations:** 1 Department of Radiology, Memorial Sloan-Kettering Cancer Center, New York, NY USA; 2 Institute of Diagnostic and Interventional Radiology, University Hospital Zurich, Raemistrasse 100, 8091 Zurich, Switzerland

**Keywords:** Computed tomography, Spleen, Hypodense lesion, Abdominal pathology

## Abstract

**Background:**

As the largest single lymphatic organ in the human body, the spleen is responsible for central immunological and haematological tasks. Therefore, the spleen can be subject to a wide range of pathologic disorders. Computed tomography (CT) represents the most widely applied cross-sectional abdominal imaging technique and is considered the imaging modality of choice for the evaluation of numerous abdominal pathological conditions. Hypodense splenic lesions are frequently encountered on abdominal CT images. Although most hypodense lesions of the spleen can be considered benign, some findings and clinical conditions warrant closer attention to the lesion. CT offers a number of morphological criteria that can be applied to differentiate hypodense lesions of the spleen, such as a the appearance of a lesion’s borders, its attenuation, as well as the presence of calcifications or solid components.

**Methods:**

This article reviews the most common splenic pathologies leading to hypodense appearances on CT images and illustrates the key CT imaging findings in the context of the clinical history of the patients.

**Conclusion:**

The key imaging findings of hypodense splenic lesions are presented in order to aid interpretation during routine evaluation of abdominal CT images.

**Teaching Points:**

• *Haemangiomas, congenital in origin, represent the most common benign lesions of the spleen.*

• *Lymphoma represents the most common malignant tumour of the, usually secondarily involved, spleen.*

• *Most hypodense splenic lesions on CT represent benign lesions that require no further work-up.*

• *For correct interpretation, hypodense splenic lesions need to be evaluated in the clinical context.*

## Introduction

Focal hypodense lesions of the spleen are frequently encountered on computed tomography (CT) images of the abdomen. Although the majority of hypodense splenic lesions do not require dedicated management or follow-up, some findings warrant closer attention [1]. Interpretation of a hypodense lesion in the spleen can be challenging, and often can only be performed correctly when certain imaging patterns are considered and the clinical history is taken into account. The purpose of this article is to review and discuss the characteristic CT imaging findings of hypodense lesions of the spleen and to illustrate selected cases in the context of the patient’s history.

## The normal spleen

Size and shape of the spleen exhibit a wide variability. While the shape is influenced by adjacent organs, determination of the normal size of the spleen can be tricky. It has been suggested, that—on transverse CT images—a maximum diameter of up to 10 cm and a perpendicular diameter of up to 6 cm can be considered normal. In adults, the craniocaudal diameter usually does not exceed 15 cm [2, 3].

**Fig. 1:**
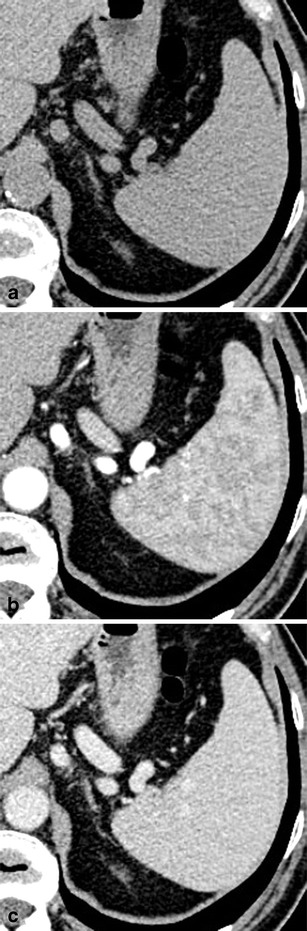
Transverse CT images acquired (**a**) before and (**b**, **c**) after the intravenous administration of iodinated contrast material in a 38-year-old man. **b** Note the trabecular enhancement pattern of the spleen during the arterial phase, when compared with the homogeneous appearance of the spleen during the (**c**) portal venous phase and on the (**a**) non-enhanced image

On non-contrast-enhanced CT images, the healthy spleen usually has a density of around 45 Hounsfield Units (HU) (Fig. 1a). A diffuse increase in splenic density can be observed in patients with haemosiderosis, sickle-cell disease and lymphoma. On contrast-enhanced, arterial-phase CT images, the spleen typically shows a heterogeneous (i.e. trabecular or serpentine) enhancement pattern due to variable flow rates of contrast-enhanced blood through the sinuses of the red pulp (Fig. 1b). Awareness of this irregular enhancement pattern is essential, because underlying pathologies or focal traumatic lesions may be obscured. On contrast-enhanced, portal venous-phase CT images, healthy splenic parenchyma has a homogenous appearance (Fig. 1c). Thus, contrast-enhanced, portal-venous phase CT images should be evaluated when searching for and interpreting lesions of the spleen.

## Cysts

Splenic cysts can be divided into true (i.e. primary) and false (i.e. secondary) cysts. True splenic cysts can be further divided into parasitic and non-parasitic (i.e. congenital and neoplastic) cysts [4]. While true cysts have epithelial lined walls, the borders of false cysts are composed of dense, fibrous tissue without an epithelial lining. It is impossible to differentiate between true and false splenic cysts by the CT imaging appearance itself. However, in clinical routine this differentiation is usually not relevant, because cysts without inherent solid components, wall-thickening or contrast-enhancement can be safely considered benign. However, it is important to note that cysts may cause complications, including infection, rupture and haemorrhage, and thus may cause clinical symptoms such as fever, upper left quadrant pain or jaundice.

### Parasitic cysts

**Fig. 2:**
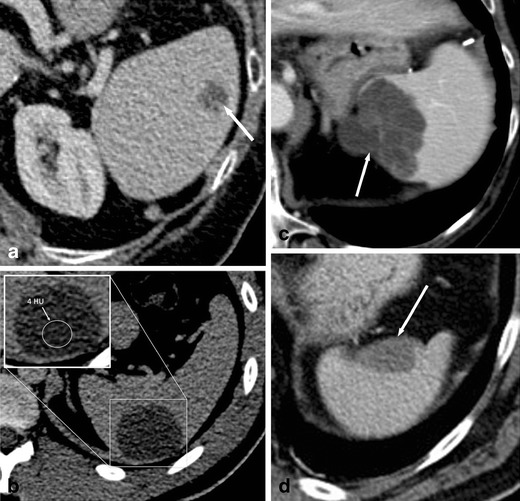
Transverse contrast-enhanced CT images acquired during the portal-venous phase illustrating the different appearances of cystic splenic lesions. **a** A 73-year-old man with hydatid disease of the spleen (*arrow*). **b** A 23-year-old man with a congenital cyst of the spleen exhibiting water-like attenuation values. **c** A 52-year-old man with a multicystic metastasis from colon cancer (*arrow*). **d** A 63-year-old man with a false cyst, presumably after trauma (*arrow*)

Parasitic cysts are caused by splenic involvement in hydatid disease, primarily due to infection with *Echinococcus granulosus* (Fig. 2a). While primary hydatid disease of the spleen is rare, secondary splenic involvement can either be caused by systemic (i.e. haematogenous) infection or subsequent to rupturing of a hepatic hydatid cyst and abdominal spread of parasites [5, 6]. Clinical manifestations of echinococcosis are rather unspecific and may include abdominal pain and fever. In case of known hepatic or pulmonary hydatid disease, a newly diagnosed cystic lesion of the spleen needs to be regarded as splenic disease involvement, especially in patients from endemic regions such as west-central Europe, Turkey, most areas of the former Soviet Union, Iran, Iraq, western and central China as well as northern Japan and the northern parts of the United States.

On contrast-enhanced CT, parasitic cysts may present as unilocular or multilocular and can be found anywhere within the spleen. Parasitic cysts are well-defined, demonstrate homogeneous fluid content with attenuation values similar to water and do not exhibit contrast-enhancement. In addition, a Waterlilly sign is frequently observed [7]. Wall calcifications may occur as parasites die. As parasitic cysts grow larger, a mass effect can lead to splenomegaly [5, 6].

### Congenital cysts

Congenital cysts account for 2.5 % of splenic cysts and are usually detected incidentally in children and young adults [8, 9]. Although they are mostly stable in size and remain asymptomatic, large cysts may cause symptoms due to a mass-effect.

On contrast-enhanced CT, congenital cysts can be unilocular or multilocular and can be found anywhere within the spleen. They appear as well-defined, thin-walled, spherical lesions with homogeneous content and water-like attenuation values around 0–10 HU [10] (Fig. 2b). Wall trabeculations or peripheral septations can be seen in up to 86 % of cases on CT and wall calcifications in up to 14 % [11]. Congenital cysts do not exhibit contrast enhancement.

### Neoplastic cysts

Neoplastic cysts may be associated with splenic metastases (Fig. 2c), lymphangiomas and haemangiomas. They usually represent true cysts. Wall thickening and adjacent solid, contrast-enhancing components facilitate the differentiation between benign and malignant cystic lesions.

### False cysts

False cysts account for approximately 80 % of splenic cysts and are also referred to as secondary cysts. They mostly originate as a consequence of trauma, infection or infarction and are thought to represent the end-stage of intraparenchymal haematomas.

On contrast-enhanced CT, false cysts can be unilocular or multilocular in appearance, are well-defined and usually located close to the capsule of the spleen. Wall calcifications are observed more commonly than in true cysts (up to 50 % of cases; Fig. 2d) [11].

## Splenic trauma

The spleen is a highly vascularised organ, receives up to 5 % of the cardiac output, and contains approximately 500 ml of blood in reserve. In blunt abdominal trauma situations, the spleen is the most commonly affected organ [4]. Due to its anatomical structure, thin capsule, ligamentous fixation and intraperitoneal location, the spleen has a large potential for uncontained haemorrhage. The risk of splenic injuries increases in case of splenomegaly. The spectrum of injuries includes subcapsular, intraparenchymal or perisplenic haematomas as well as lacerations, ruptures and infarctions due to vascular injuries, as well as contained vascular injuries such as pseudoaneurysm and arteriovenous fistula [12]. In severe trauma, the spleen can rupture and multiple, devascularised splenic fragments can be seen in the left upper quadrant of the abdomen. Usually, splenic ruptures are associated with extensive intraperitoneal haemorrhage. Traditionally, splenic injuries are classified according to the splenic injury grading system of the American Association for the Surgery of Trauma [13]. In addition, multiple CT grading systems had been proposed by various authors [14], as contrast-enhanced CT is an excellent imaging modality for the assessment of traumatic lesions of the spleen [15]. However, care needs to be taken not to misinterpret congenital anomalies of the spleen such as lobulations or splenic clefts for splenic injuries.

**Fig. 3:**
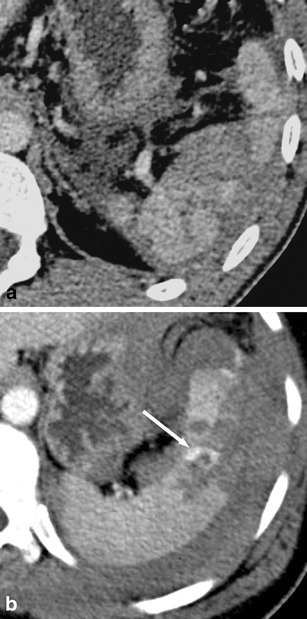
Transverse contrast-enhanced CT images acquired during the late arterial phase. **a** A 23-year-old man who was involved in a motorcycle accident and suffered a splenic laceration with extensive intraparenchymal, subcapsular and perisplenic haematoma. **b** A 34-year-old woman who was involved in a motor vehicle accident and suffered a splenic laceration. Note the active contrast extravasation within the spleen (*arrow*)

On non-contrast-enhanced CT images, haematomas can present as either intraparenchymal, subcapsular or perisplenic fluid collections with increased density (>30 HU; Fig. 3a) [12]. Extravasation of contrast material can sometimes be seen on contrast-enhanced CT images (Fig. 3b).

On contrast-enhanced CT images, splenic lacerations mostly present themselves as ill-defined, irregular, geographic hypodense areas within the spleen due to a lack of perfusion with contrast material. Lacerations can be located anywhere in the spleen (i.e. centrally, close to the hilum, subcapsular). On non-contrast-enhanced CT images, the density of lacerations is usually higher than the density of the normal spleen due to haemorrhage. Large lacerations or intraparenchymal haematomas can cause a mass effect, which is also responsible for clinical symptoms including left upper quadrant pain.

## Splenic abscess

The prevalence of splenic abscesses during autopsy had been reported between 0.14 % and 0.7 % [16]. Over the last two decades, the incidence of splenic abscesses has increased due to the wide-spread use of immunosuppression in patients with organ transplantation, the more common use of chemotherapy and corticosteroids as well as a greater incidence of AIDS. Splenic abscesses include pyogenic, fungal and tuberculous abscesses.

**Fig. 4:**
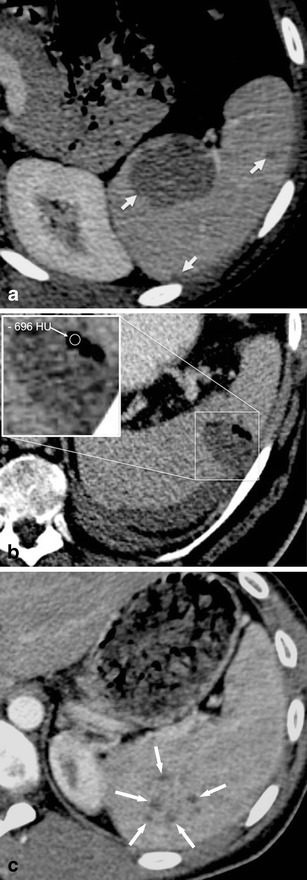
Transverse contrast-enhanced CT images acquired during the portal-venous phase. **a** A 19-year-old woman with multiple pyogenic splenic abscesses during a period of immunosuppression and haematogenous spread of *Staphylococcus aureus* (*short arrows*). **b** A 48-year-old man with a pyogenic splenic abscess exhibiting gas formations and subcapsular fluid accumulation due to a spontaneous rupture of the abscess. **c** A 27-year-old man with multiple tuberculous abscesses (*long arrows*)

Pyogenous abscesses are caused by either haematogenous spread of bacteria (i.e. *Staphylococcus*, *Streptococcus*, *Escherichia coli* or *Salmonella*), penetrating trauma, prior splenic infarction or contiguous infection (i.e. from perinephric abscess or pancreatitis) [17, 18]. Most pyogenic abscesses are unilocular, but can be multifocal in up to 26 % of cases [18–21]. On contrast-enhanced CT images, pyogenic abscesses typically exhibit a “rim-enhancement” of the outside-facing portion of the abscesses’ wall (Fig. 4a). The inside-facing portions of the wall usually show less-enhancing or non-enhancing components, which represent fibrous and proteinaceous material. The content of a pyogenic abscess usually appears inhomogeneous with density values ranging from 20 to 40 HU. Gas formations within the abscess can be encountered and usually confirm the diagnosis of a pyogenic abscess (Fig. 4b).

Fungal abscesses are mostly multifocal (i.e. up to 90 %), smaller than pyogenic abscesses (<2 cm), and do not show rim enhancement on contrast-enhanced CT images. Due to their small size, lack of contrast enhancement and miliary pattern, fungal abscesses can be difficult to detect on CT images. Therefore, CT cannot reliably rule out fungal abscesses, as enlargement of the spleen remains the only imaging finding at times.

Tuberculous abscesses of the spleen, usually confirmed during autopsy [22, 23], can sometimes be seen in patients with severe, disseminated tuberculous disease. However, in patients with known pulmonary tuberculosis, the presence of multiple, small, hypodense, splenic lesions on contrast-enhanced CT needs to be regarded as abdominal tuberculous spread, unless proven otherwise. Tuberculous abscesses are usually small (<2 cm), ill-defined and can show mild contrast-enhancement (Fig. 4c) [24].

## Sickle cell disease

**Fig. 5:**
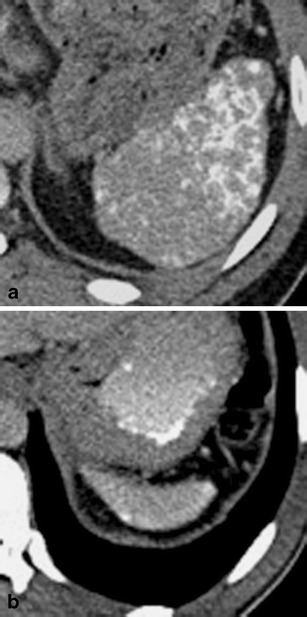
Transverse contrast-enhanced CT images acquired during the portal venous phase. **a** A 43-year-old man with advanced sickle-cell disease. Note the irregular shape of the spleen, as well as the increased density of the splenic parenchyma, together with extensive calcifications as a consequence of constantly occurring micro-infarctions. **b** A 38-year-old man with sickle-cell disease exhibiting end-stage splenic involvement. Note the increased density, calcifications of the shrunken spleen

While passing through the sinuses of the red pulp, the blood’s haematocrit increases by approximately 60 % due to plasma removal. In addition, the blood flow decelerates to allow for maximal interaction of antigenic material and structurally abnormal erythrocytes with macrophages and reticuloendothelial cells. In order to survive this process, erythrocytes need to be deformable in order to pass through tiny slits between endothelial cells. In patients with sickle cell disease, erythrocytes are rigid and frequently occlude the small sinuses of the red pulp. This leads to micro-infarctions and micro-haemorrhage, which then become visible on contrast-enhanced CT as small, disseminated hypodense, ill-defined lesions. In the early course of the disease, the spleen can be enlarged. As the disease advances, the spleen loses more and more of its function, shrinks and may become calcified (Fig. 5). Therefore, in patients with sickle cell disease, the presence of multiple small hypodense splenic lesions is strongly suggestive of sickle cell-induced splenic infarctions.

## Infarction

**Fig. 6:**
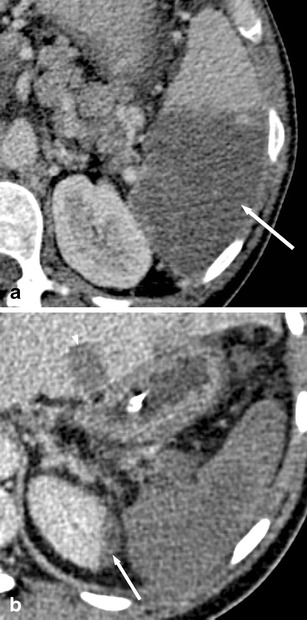
Transverse contrast-enhanced CT images acquired during the portal-venous phase. **a** A 49-year-old woman with a large splenic infarction secondary to thromboembolism from atrial fibrillation. **b** A 42-year-old woman with a complete splenic infarction, partial renal infarction (*arrow*) and hepatic infarction (*arrowhead*) due to a cardiogenic shock caused by sudden cardiac arrest

Splenic infarction is caused by a lack of perfusion of either certain regions of the spleen (Fig. 6a) or the entire organ (Fig. 6b). The most common event leading to infarctions of the spleen is partial or total occlusion of the splenic artery or its branches. Patients typically present with left upper quadrant abdominal pain, but without fever. Large splenic infarctions are usually caused by thromboembolic conditions mostly originating from atrial fibrillation, whereas micro-infarctions can be seen in patients with sickle cell anaemia, lymphoma and leukaemia.

On contrast-enhanced CT images, splenic infarctions present as wedge shaped, hypodense areas with the base facing the splenic capsule. Enhancement of the affected region can be observed on delayed-phase images. Splenic infarctions can cause a mass effect and may present as ill-defined areas during early stages and well-defined areas during late stages. The development of secondary abscesses or splenic rupture and subsequent haemorrhage represent potential complications secondary to splenic infarction.

## Hamartoma

Hamartomas of the spleen, also referred to as splenomas, are benign and typically asymptomatic lesions which are often discovered incidentally on imaging. They can be associated with tuberous sclerosis and Wiskott-Aldrich like syndrome. Hamartomas do not possess a capsule. The histological structure consists, depending on the type, of a mixture of either red or white pulp elements.

**Fig. 7:**
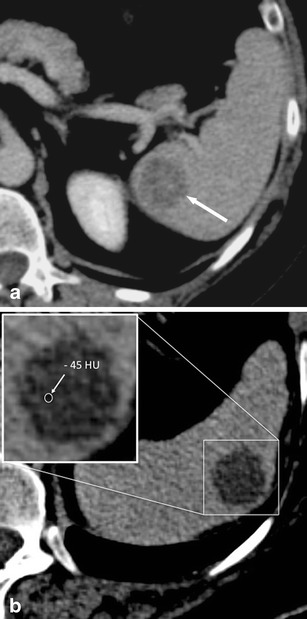
Transverse contrast-enhanced CT images acquired during the portal-venous phase. **a** A 54-year-old woman with a hamartoma that exhibits mild contrast enhancement. **b** A 76-year-old woman with a hamartoma that presents with focal areas of fat attenuation

On contrast-enhanced CT, hamartomas appear as solitary, well-defined, solid lesions that can show prolonged contrast-enhancement due to stagnant blood flow within the sinuses of the red pulp (Fig. 7a) [25–27]. Calcifications, central areas of necrosis as well as macroscopic fat may be observed. Fat can be detected on CT as areas with negative attenuation (Fig. 7b). The prolonged enhancement on CT assists in differentiating hamartomas from malignant tumours of the spleen [25, 28].

## Haemangioma

Haemangiomas account for the most common primary benign splenic neoplasm, with a reported autopsy prevalence of up to 14 % [29]. Multiple haemangiomas can be observed predominantly in systemic angiomatosis patients (i.e. Beckwith-Wiedemann, Klippel-Trenauney-Weber and Turner syndromes). Haemangiomas, congenital in origin, consist of vascular channels filled with slow-flowing blood.

**Fig. 8:**
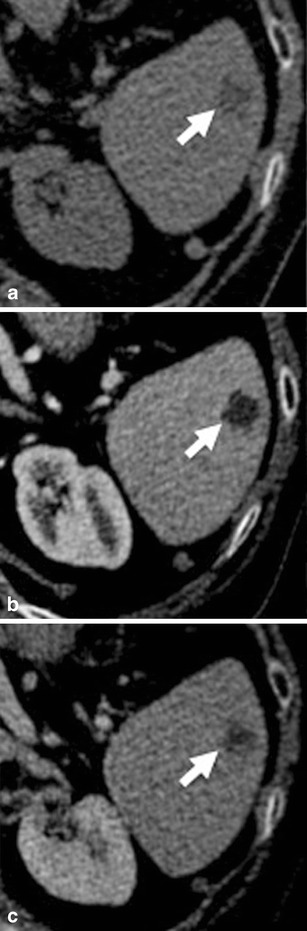
Transverse CT images acquired (**a**) before and (**b**, **c**) after intravenous administration of iodinated contrast material in a 74-year-old woman with a splenic haemangioma. Note the typical nodular enhancement beginning in the arterial phase and extending in a centripetal manner during the portal-venous phase

On non-contrast-enhanced CT images, most haemangiomas are isodense or hypodense when compared with normal splenic parenchyma. After the administration of contrast material, haemangiomas typically exhibit early, peripheral nodular enhancement, which extends towards the centre of the well-defined lesion over time [27, 30, 31] (Fig. 8). However, the enhancement pattern can differ especially in cavernous haemangiomas due to the presence of fibrosis and micro-infarctions. Due to the variable enhancement pattern and differences in timing during the administration of contrast material, haemangiomas may appear slightly larger or smaller on follow-up CT images at times. The most important—though very rare—complication of splenic haemangiomas is their spontaneous rupture, which can result in extensive intraperitoneal haemorrhage.

## Lymphangioma

Lymphangiomas of the spleen are usually detected incidentally in children and young adults. They are typically located close to the splenic capsule and may be unilocular or multilocular in appearance.

**Fig. 9:**
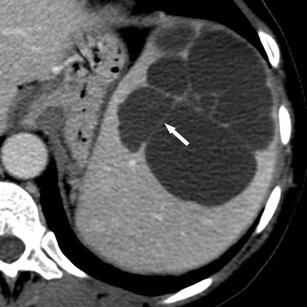
Transverse contrast-enhanced CT image acquired during the portal-venous phase in a 38-year-old woman illustrating a lymphangioma of the spleen. Note the lesion’s septations (*arrow*) that may enhance slightly after the administration of intravenous contrast material and homogeneous water-like content in the absence of solid components. The most important differential diagnosis to consider in this case would be a hydatid (echinococcal) cyst

On contrast-enhanced CT images, lymphangiomas present as well-defined lesions. The walls of the vascular channels, which comprise splenic lymphangiomas, are thin, and contrast-enhancing components are not identified on CT [32] (Fig. 9). Focal scars may be present. Lymphangiomas are congenital malformations, which can become life-threatening in children with lymphangiomatosis. However, isolated splenic lymphangioma is very uncommon in adults. Large lymphangiomas can become symptomatic due to a mass effect and splenomegaly.

## Sarcoidosis

Sarcoidosis is a granulomatous systemic disease of unknown aetiology that can affect numerous organs and locations, infrequently involving the spleen.

**Fig. 10:**
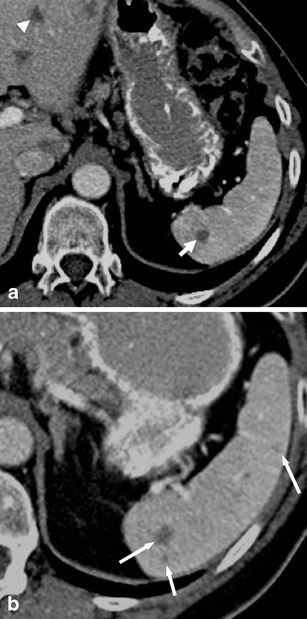
**a**, **b** Transverse contrast-enhanced CT images acquired during the portal-venous phase at different levels in a 55-year-old woman with sarcoidosis affecting the liver (*arrowhead*) and the spleen (*short arrow*, *long arrows*)

On contrast-enhanced CT, the most important finding in patients with sarcoidosis is splenomegaly. However, small hypodense, ill-defined lesions with contrast-enhancement may also be encountered (Fig. 10). Usually the granulomas do not enhance more than healthy spleen; however, their attenuation is higher than that of splenic cysts. Lymphadenopathy in the splenic hilum is an important adjacent finding that often suggests the diagnosis of splenic sarcoidosis.

## Peliosis

Peliosis of the spleen is rare, since the liver is usually the primary organ affected by this disease. Peliosis is usually found incidentally on diagnostic imaging or during autopsy, as most patients remain asymptomatic [33]. However, spontaneous splenic rupture had been reported as a potential complication [34]. Histopathologically, dilated sinusoids as well as fluid- and blood-filled cavities can be found within the splenic parenchyma in patients with peliosis [35].

**Fig. 11:**
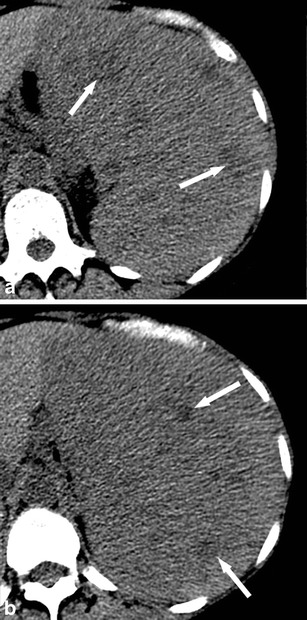
**a**, **b** Transverse non-contrast-enhanced CT images acquired at two different levels in a 55-year-old woman with splenic peliosis, exhibiting multiple hypodense lesions of different size (*arrows*) within a massively enlarged spleen

On contrast-enhanced CT, peliosis exhibits multiple, small, ill-defined, hypodense lesions (Fig. 11). However, due to haemorrhage, peliosis lesions can present hyperdense as well. Different patterns of enhancement have been reported including early peripheral enhancement with delayed centripetal enhancement, similar to haemangiomas [36].

## Littoral cell angioma

Littoral cell angiomas represent rare vascular tumours that arise from the cells that line the red pulp. They are usually detected incidentally, since most littoral cell angiomas remain asymptomatic. While the majority of littoral cell angiomas is benign, cases of malignant lesions have been reported [37]. More importantly, associations with colorectal carcinoma, renal cell carcinoma, hepatocellular carcinoma, lung cancer, lymphoma and pancreatic adenocarcinoma as well as meningioma have been reported [38].

**Fig. 12:**
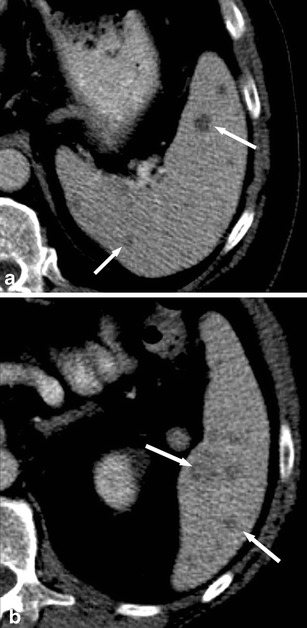
**a**, **b** Transverse contrast-enhanced CT images acquired during the portal-venous phase at two different levels in a 55-year-old man with littoral cell angioma, which presents as multiple hypodense, partially contrast-enhancing, lesions of different size (*arrows*)

On contrast-enhanced CT images, littoral cell angiomas usually present as multiple, ill-defined, hypodense lesions that show prolonged contrast-enhancement due to their histological structure of multiple vascular channels [39–41] (Fig. 12).

## Lymphoma

Lymphoma represents the most common malignant tumour of the spleen [42, 43]. Isolated primary splenic lymphoma represents less than 2 % of all lymphomas [44]. In patients with lymphoma, the spleen is usually involved secondarily.

**Fig. 13:**
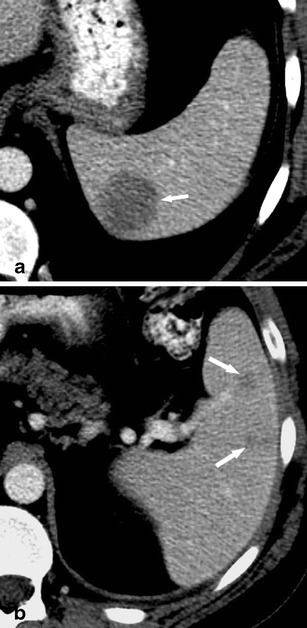
**a**, **b** Transverse contrast-enhanced CT images acquired during the portal-venous phase at two different levels in a 55-year-old man with diffuse large B-cell lymphoma exhibiting multiple hypodense, mildly contrast-enhancing lesions within the spleen (*arrows*); with the latter usually being enlarged

On contrast-enhanced CT images, it may be challenging to differentiate lymphoma nodules from sarcoidosis or fungal abscesses, which frequently occur in lymphoma patients due to immunodeficiency. However, in such cases, the presence of hilar lymphadenopathy is suggestive of splenic lymphoma [45], but should not be confused with sarcoidosis. Lymphoma of the spleen can present as splenomegaly without focal lesions, multiple small (<1 cm) or large (<10 cm) lesions, or a single solitary lesion (Fig. 13). The diagnosis of splenic lymphoma can be most easily made in the context of the clinical history and in knowledge of the disease.

## Metastases

Metastases to the spleen usually occur in the context of multi-visceral, metastatic end-stage cancer [46]. Breast, lung, ovarian, colorectal and gastric carcinomas, as well as skin melanoma, are considered the most common primary sources [47], with skin melanoma accounting for the most aggressive type of splenic involvement, affecting up to 30 % of patients [48, 49]. It is important to note that metastases from skin melanoma can present as hypodense on non-contrast-enhanced CT images.

**Fig. 14:**
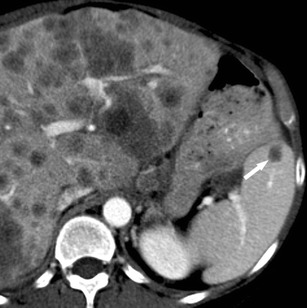
Transverse contrast-enhanced CT image acquired during the portal-venous phase in a 47-year-old woman with metastatic ovarian cancer. The newly diagnosed hypodense lesion within the spleen (*arrow*) needs to be regarded as a metastasis unless proven otherwise

On contrast-enhanced CT images, hypodense, ill-defined, contrast-enhancing lesions in patients with known malignancies need to be regarded as splenic metastases until proven otherwise. Metastases may, depending on the primary tumour, appear either hypodense or hyperdense (Fig. 14) during the portal-venous phase and can show cystic components.

## Angiosarcoma

Splenic angiosarcomas are rare but very aggressive tumours. Either solitary or multiple, contrast-enhancing lesions can be seen on CT [50–52]. The enhancement can be similar to that of haemangiomas. An early onset of metastasis, predominantly to the liver, is observed.

## Conclusions

In general, the majority of hypodense splenic lesions on contrast-enhanced, portal-venous CT represent benign lesions that require no further work-up. However, certain imaging appearances—such as ill-defined lesion borders, presence of solid, contrast-enhancing components and increased attenuation of the lesion—must draw the attention of the radiologist to a potentially more relevant disease. For correct interpretation, hypodense lesions of the spleen need to be evaluated in the clinical context of the history, because CT imaging appearances of certain entities are overlapping and often cannot be distinguished with certainty (Table 1). In the presence of a newly diagnosed, hypodense lesion of the spleen on contrast-enhanced, portal-venous CT, the answers to the following questions can help in clarifying the diagnosis: Is there a known primary tumour? Does the patient have any other signs of metastatic disease? Does the patient suffer from sarcoidosis or tuberculosis? Does the patient have fever? Is there a recent history of trauma?

**Table 1:** Differential CT imaging features of hypodense lesions of the spleen

	Distribution	Number of lesions	Margin	Mass effect	Contrast enhancement	Spleen size	Patient history
Parasitic cyst	Random	Solitary	Well-defined	None	None	Normal	Hydatid disease (lung, liver)
		Multiple					
Congenital cyst	Random	Solitary	Well-defined	Possible	Trabeculae	Normal	–
Neoplastic cyst	Random	Solitary	Well-/ill-defined	None	Yes	Normal	Primary tumour
		Multiple					
False cyst	Subcapsular	Solitary	Well-defined	None	None	Normal	Past splenic trauma
Traumatic injury	Random	Solitary	Ill-defined	Possible	None	Normal	Trauma
		Multiple					
Pyogenic abscess	Random	Solitary	Ill-defined	Possible	Rim enhancement	Normal	Infectious condition (localised/systemic)
		Multiple					
Sickle cell disease	Random	Multiple	Ill-defined	None	None	Normal (early stage)	Sickle cell disease
						Small (late stage)	
Infarction	Oriented towards capsule	Solitary	Well-/ill-defined	None	Prolonged	Normal	Left upper quadrant pain
		Multiple					Thromboembolic disease
Hamartoma	Random	Solitary	Well-defined	None	Prolonged	Normal	Mostly incidental finding; tuberous sclerosis, Wiskott-Aldrich-like syndrome
Haemangioma	Random	Solitary	Well-/ill-defined	None	Centripetal	Normal	Mostly incidental finding; Beckwith-Wiedemann, Klippel-Trenauney-Weber, Turner syndrome
		Multiple					
Lymphangioma	Subcapsular	Solitary	Well-defined	Possible	Trabeculae	Normal	Incidental finding in child, young adult
		Multiple					
Sarcoidosis	Random	Multiple	Ill-defined	Possible	Yes	Enlarged	Sarcoidosis
Peliosis	Random	Multiple	Ill-defined	Yes	Similar to haemangioma	Enlarged	Peliosis hepatis
Littoral cell angioma	Random	Multiple	Ill-defined	None	Prolonged	Normal	Colorectal carcinoma, renal cell carcinoma, hepatocellular carcinoma, lung cancer, lymphoma, pancreatic carcinoma
Lymphoma	Random	Solitary	Ill-defined	Yes	Mild	Enlarged	Associated lymphadenopathy; mostly patients with clinically known lymphoma
		Multiple					
Metastases	Random	Solitary	Ill-defined	Possible	Yes	Normal	Breast, lung, ovarian, colorectal and gastric carcinomas; skin melanoma
		Multiple				Enlarged	
Angiosarcoma	Random	Solitary	Ill-defined	Possible	Yes	Normal	Mostly discovered due to early onset of distant metastases
		Multiple					
